# Binding of Sulpiride to Seric Albumins

**DOI:** 10.3390/ijms17010059

**Published:** 2016-01-04

**Authors:** Viviane Muniz da Silva Fragoso, Carla Patrícia de Morais Coura, Luanda Yanaan Hoppe, Marília Amável Gomes Soares, Dilson Silva, Celia Martins Cortez

**Affiliations:** 1 Laboratory of Innovations in Therapies, Education and Bioproducts, Oswaldo Cruz Institute/FIOCRUZ, Av. Brasil 4365, Rio de Janeiro 21045-900, Brazil; luanda.hoope@ioc.fiocruz.br; 2 Postgraduation in Medical Sciences, Rio de Janeiro State University, Av. Manoel de Abreu, 444, Rio de Janeiro 20550-171, Brazil; carla.morais@inca.gov.br (C.P.M.C.); dilsons@uerj.br (D.S.); ccortezs@ime.uerj.br (C.M.C.); 3 Applied Mathematics, Rio de Janeiro State University, Rua São Francisco Xavier, 524, Rio de Janeiro 20559-900, Brazil; marilia.agsoares@gmail.com

**Keywords:** sulpiride, interaction, albumins, fluorescence quenching

## Abstract

The aim of this work was to study the interaction of sulpiride with human serum albumin (HSA) and bovine serum albumin (BSA) through the fluorescence quenching technique. As sulpiride molecules emit fluorescence, we have developed a simple mathematical model to discriminate the quencher fluorescence from the albumin fluorescence in the solution where they interact. Sulpiride is an antipsychotic used in the treatment of several psychiatric disorders. We selectively excited the fluorescence of tryptophan residues with 290 nm wavelength and observed the quenching by titrating HSA and BSA solutions with sulpiride. Stern-Volmer graphs were plotted and quenching constants were estimated. Results showed that sulpiride form complexes with both albumins. Estimated association constants for the interaction sulpiride–HSA were 2.20 (±0.08) × 10^4^ M^−1^, at 37 °C, and 5.46 (±0.20) × 10^4^ M^−1^, at 25 °C. Those for the interaction sulpiride-BSA are 0.44 (±0.01) × 10^4^ M^−1^, at 37 °C and 2.17 (±0.04) × 10^4^ M^−1^, at 25 °C. The quenching intensity of BSA, which contains two tryptophan residues in the peptide chain, was found to be higher than that of HSA, what suggests that the primary binding site for sulpiride in albumin should be located next to the sub domain IB of the protein structure.

## 1. Introduction

Sulpiride is an antipsychotic drug of the benzamine class, which presents strong chemical and clinical similarity with amisulpride, and it is used in the treatment of a large range of psychotic disorders [1,2]. About 1% of the general population is affected by psychotic diseases with high prevalence (16%) among people with familial history of schizophrenia [3,4].

Antipsychotic drugs have been traditionally classified into typical and atypical, where the so called typical antipsychotics were the first developed drugs. Typicals have great efficacy on positive symptoms but present low response in the case of negative symptoms. Atypical antipsychotics, such as risperidone, are the second-generation antipsychotic drugs which are efficient to reduce both positive and negative symptoms, without important prejudice of physiological effects of dopamine via nigro-striatal. Atypicals present an important safety advantage over typicals because of their low potential to generate extrapyramidal effect and tardive dyskinesia [5].

Sulpiride is a selective post-synaptic dopamine D2 antagonist [2], and there are suggestions that it could be regarded as an atypical antipsychotic [6,7,8] because of its clinical benefits to minimize negative defective symptoms, as well as its partial activity against positive symptoms [9,10]. In addition, sulpirides have fewer extrapyramidal effects than other typical antipsychotics such as haloperidol [1]. Lai *et al.* [11] have shown, using pragmatic outcome measures, that the effectiveness of sulpiride was better than haloperidol and risperidone. The most troubling side effects of sulpiride were weight gain and endocrine-related symptoms, which may also affect patient persistence [4].

A meta-analysis study has concluded that atypical anti-psychotics comprise a heterogeneous group of drugs presenting different pharmacological properties, such as mechanisms of action, therapeutic efficacy and side effects besides cost-effective profile [12]. In such a context, we regard as questionable whether sulpiride is properly classified as an atypical antipsychotic.

It is known that the ratio of the binding of sulpiride to plasmatic proteins is about 40% [13]. The drug-protein binding is a very important pharmacokinetic parameter, since the balance between free and combined drug portions in plasma is a determinant factor for the intensity and the duration of their clinical effects, distribution and elimination [14,15].

In an earlier study [16], we used the spectrofluorimetric binding analysis [17] to explore the interaction of risperidone with albumins. Results showed that this drug quenches the fluorescence of albumin and forms complexes with both human serum albumin (HSA) and bovine serum albumin (BSA).

The present paper reports results produced by the spectrofluorimetric study of the interaction of sulpiride with HSA and BSA, and comparing data obtained for the two albumins, we enhanced possibilities to better identify and characterize the binding region, presenting a mathematical model to estimate the quenching of albumins by fluorescent drugs.

## 2. Results and Discussion

### 2.1. Fluorescence Quenching

**Figure 1:**
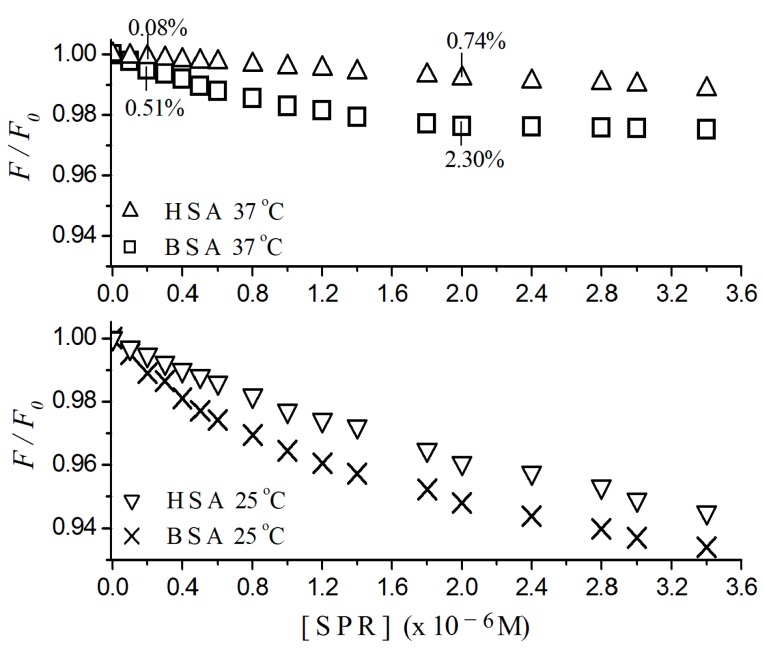
Normalized fluorescence quenching plots of human serum albumin (HSA) and bovine serum albumin (BSA) titrated with sulpiride at 25 and 37 °C. Excitation λ = 290 nm; [albumin] = 2 × 10^−6^ M; 10^−3^ M phosphate buffer, pH 7.4. [SPR] = sulpiride concentration.

HSA and BSA solutions were titrated by sulpiride at 25 and 37 °C and plots of fluorescence quenching, Stern-Volmer constants, binding constants and binding sites were shown. Figure 1 shows the normalized fluorescence quenching plots for HSA and BSA titrated by sulpiride related to the sulpiride concentration, at 37 °C, Figure 1a and at 25 °C, Figure 1b. Each point in the plot represents the average of three experiments, within standard deviations lower than 10%, ever considering four points around the maximum emission, λ_max_ = 340 nm for BSA, and λ_max_ = 338 nm for HSA. The values of F/F_0_ were corrected by Equation (3). For the molar ratio sulpiride/HSA and sulpiride/BSA of 1:10 at 37 °C, the ligand quenches 0.08 (±0.03)% and 0.51 (±0.10)% of HSA and BSA fluorescence, respectively. Otherwise, for the molar ratio 1:1 at the same conditions, the quenching increased to 0.74 (±0.10)% and 2.30 (±0.20)% for HSA and BSA, respectively.

The study of the interaction of sulpiride with albumin has great relevance once this protein represents 60% of the total plasmatic proteins and plays a crucial role in transport hydrophobic ligands [14,18,19].

Despite the exclusive use of sulpiride in humans, we also estimated spectrofluorimetric data for its interaction with BSA, once such comparative analysis allows us to verify the approximate localization of the primary binding site for HSA and BSA in albumins. The HSA molecule only has a single tryptophan residue located in sub domain IIA (tryptophan 214), while BSA contains another residue, in sub domain IB (tryptophan 134), in addition to that tryptophan residue 212 in subdomain IIA [20,21,22,23].

Sulpiride has quenched the fluorescence of both albumins just after the first additions (Figure 1), although the intensity of the fluorescence emission of BSA is stronger than the fluorescence emitted by HSA, due to its two tryptophan residues. The quenching of the BSA fluorescence by sulpiride was more intense than that of HSA, especially at 37 °C and even at low concentrations (<0.6 × 10^−6^ M) of the drug. That suggests that sulpiride suppresses more intensively the emission of the tryptophan residue 134 than the emission of tryptophan residue 212, indicating that the affinity sites to sulpiride in albumins are closer to sub domain IB (or within this) than the sub domain IIA.

### 2.2. Stern-Volmer Graphs of Albumins Titrated with Sulpiride

**Figure 2:**
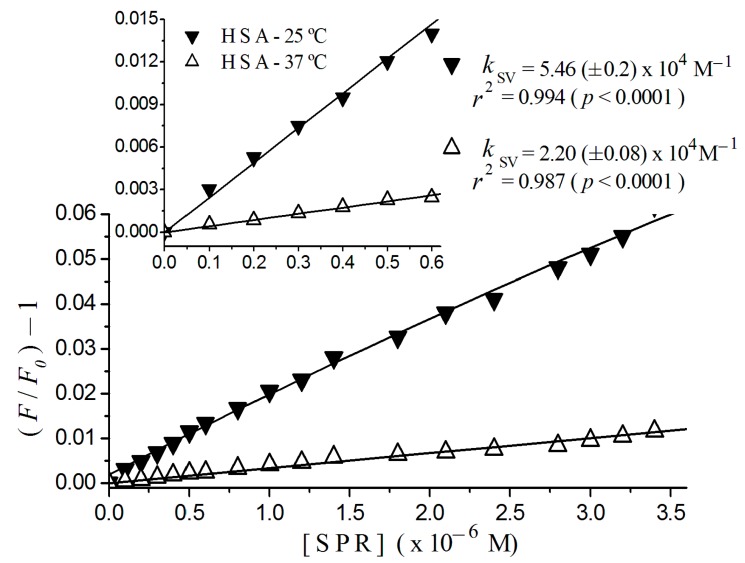
Stern-Volmer plots of HSA titrated with sulpiride at 25 and 37 °C. Excitation λ= 290 nm; (albumin) = 2 × 10^−6^ M; 10^−3^ M phosphate buffer, pH 7.4, [SPR] = sulpiride concentration. Detail shows plot region for low sulpiride concentration region with regression coefficient and Stern-Volmer constant estimated for this region.

In Figure 2, it is possible to observe the linear tendency of Stern-Volmer plots of HSA at 25 and 37 °C. The detail of this figure shows plots for (sulpiride) <0.6 × 10^−6^ M with respective constants, 2.20 (±0.08) × 10^4^ M^−1^, at 37 °C, and 5.46 (±0.20) × 10^4^ M^−1^, at 25 °C. It also presents regression coefficients, *r*^2^ = 0.984 at 37 °C and *r*^2^ = 0.994 at 25 °C for *p* < 0.0001.

**Figure 3:**
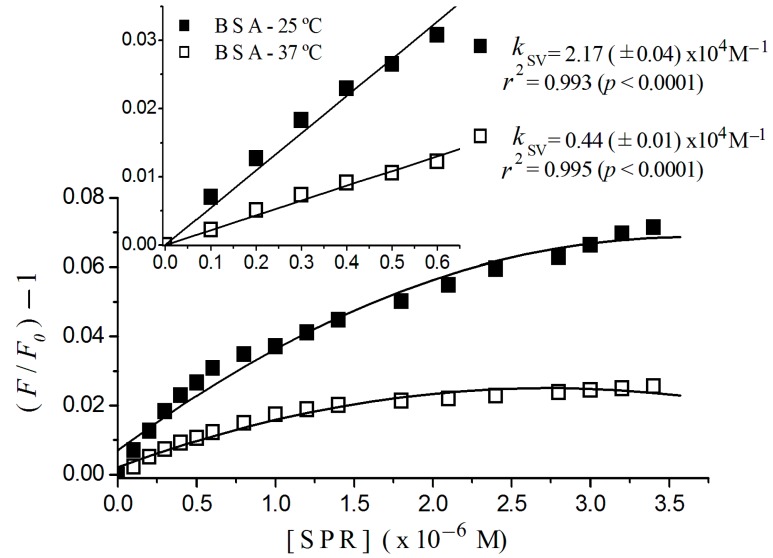
Stern-Volmer plots of BSA titrated with sulpiride at 25 and 37 °C. Excitation λ = 290 nm; (albumin) = 2 × 10^−6^ M; 10^−3^ M phosphate buffer, pH 7.4, [SPR] = sulpiride concentration. Detail shows plot region for low sulpiride concentration region, with regression coefficient and Stern-Volmer constant estimated for this region.

Figure 3 shows plots for BSA, where it was possible to fit a linear adjust to sulpiride concentrations lower than 0.6 × 10^−6^ M, with regression coefficients of 0.995 at 37 °C, and 0.993 at 25 °C for *p* < 0.0001. From an overall view, we can see a downward concave curvature toward the *x*-axis for sulpiride for concentrations higher than 0.6 × 10^−6^ M. In the figure inset, we can see Stern-Volmer constants of 0.44 (±0.01) × 10^4^ M^−1^, at 37 °C and 2.17 (±0.04) × 10^4^ M^−1^, at 25 °C.

In sequence, to calculate constants and to define the quenching mechanism, we have plotted the Stern-Volmer graphs, as shown in Figure 2. The linear tendency (*r*^2^ > 0.996, *p* < 0.0001) of the plot of sulpiride–HSA for increasing concentrations, is compatible to the existence of one single binding site to sulpiride in the proximity of the residue of tryptophan, combined to the occurrence of a single type of quenching [17].

Otherwise, the nonlinearity of the Stern-Volmer plot for BSA shown in Figure 3 confirms that the two tryptophan-residues in BSA are not equally accessible to sulpiride. The concave curvature toward the *x*-axis in addition suggests that other binding sites could have been exposed as the drug concentration increased [17].

Examining the influence of the temperature on the Stern-Volmer plot for HSA (Figure 2) and BSA (Figure 3), we can observe that an increase of 12 °C in the temperature causes a quenching decreasing, pointing to the occurrence of static quenching, which means the formation of a complex sulpiride-albumin. In that case, the Stern-Volmer constants estimated here can be named association constants [17].

In previous work [16], we found the presence of a high affinity binding site for risperidone in albumins next to or in the neighboring of sub domain IB, where the ligand binds forming the complex risperidone-albumin. The formation of a complex drug-HSA has been also found to occur with other antipsychotic drugs, such as clozapine [24], and phenotiazines: tioridazine, triflupromazine, and levomepromazine [25,26]. On the other hand, Silva *et al.* showed that the phenotiazine clorpromazine interacts with HSA without forming a complex [27].

HSA has two hydrophobic cavities located in subdomains II and III, which are considered primary sites for high affinity binding to the majority of drugs. Anionic heterocyclic compounds of large molecular size bind especially in the site Sudlow I, located in subdomain IIA. The site II (in subdomain IIIA) presents higher affinity to aromatic carboxylic drugs in therapeutic concentration, being pointed out as one of the principal binding sites for these kinds of compounds. However, low affinity and selectivity sites are also involved, for higher concentrations [28,29,30].

### 2.3. Fluorescence Quenching of HSA Titrated with Antipsychotics

**Figure 4:**
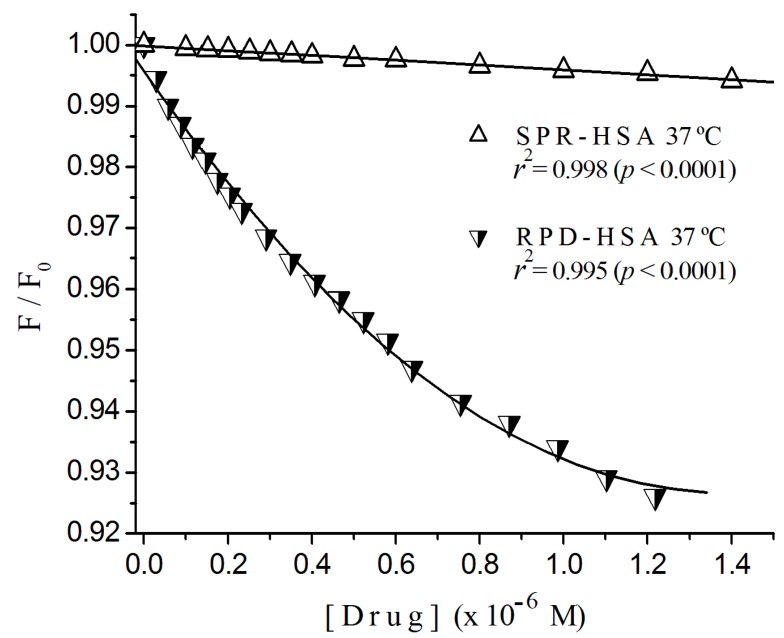
Normalized fluorescence quenching plots of HSA titrated with sulpiride and risperidone at 37 °C. Excitation λ = 290 nm; (albumin) = 2 × 10^−6^ M; 10^−3^ M phosphate buffer, pH 7.4.

Figure 4 illustrates the normalized fluorescence quenching plots of HSA by risperidone [16] and sulpiride at 37 °C, where we can see the great difference in the quenching rate for both ligands. In Table 1, we resume and compare results for HSA interacting with both ligands at 25 and 37 °C. It shows the ratio of Stern-Volmer constants and the type of the quenching, found to be static. The constant ratios risperidone/sulpiride at 37 and 25 °C were 6.5 and 4.6, respectively.

Comparing *K*_sv_ values from Table 1, we can see that *K*_sv_ for the interaction risperidone-HSA at 25 °C (2.56 (±0.09) × 10^5^ M^−1^) to that of sulpiride, 5.46 (±0.20) × 10^4^ M^−1^ we found a ratio of about 4.6-fold. By the other hand, at 37 °C, the ratio of constants, 1.43 (±0.05) × 10^5^ M^−1^ and 2.20 (±0.08) × 10^4^ M^−1^ was about 6.5-fold. These comparisons point to the occurrence of conformational changes in albumin chains which expose fluorophore groups and/or open new sites.

**Table 1:** Stern-Volmer Constant for risperidone and sulpiride with human serum albumin (HSA).

Drug	Quenching	Stern-Volmer Constant—*K*_SV_ (M^−1^)	
		37 °C	25 °C
Sulpiride	Static	2.20 (±0.08) × 10^4^	5.46 (±0.20) × 10^4^
Risperidone *	Static	1.43 (±0.05) × 10^5^	2.56 (±0.09) × 10^5^
Risperidone/Sulpiride	-	6.5	4.6

* Fragoso *et al.* [17].

### 2.4. Binding Constant and the Number of Binding Sites

**Figure 5:**
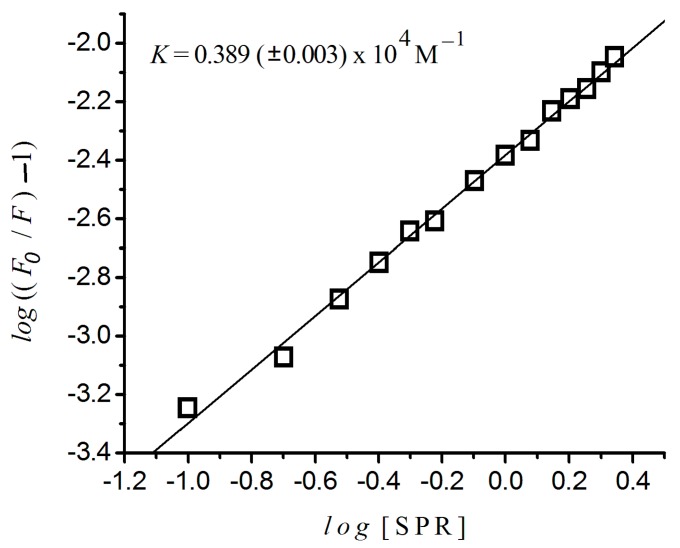
Variation of log((F_0_/F) − 1) *versus* log((sulpiride)) for HSA at 37 °C. Excitation λ = 290 nm; (albumin) = 2 × 10^−6^ M; 10^−3^ M phosphate buffer, pH 7.4.

Figure 5 presents the variation of $log(\frac{F_{0}}{F}-1)$ against the *log*([*sulpiride*]) for HSA at 37 °C, estimated by means of Equation 1. It shows a linear plot (*r*^2^ = 0.998, *p* < 0.0001) with binding constant of 0.389 (±0.003) × 10^4^ M^−1^, revealing one single binding site. They were estimated from the interception and the inclination of this plot, respectively, and these values are presented in Table 2, where, for comparison purposes, we included results of risperidone obtained from [16]. The *n* values shown in this table point to the existence of only one independent binding site for sulpiride in HSA, at 37 °C.

In addition, Table 2 provides evidence that binding constant, *K*, for risperidone-HSA (7.46 (±0.01) × 10^4^ M^−1^) [16] was many times higher than *K* for sulpiride–HSA (3.89 (±0.03) × 10^3^ M^−1^), Figure 5. Observing the *K* values, we can evaluate the distribution of a drug by the plasma. A small *K* value corresponds to a weak binding of a drug to albumin, and it means a short life time or poor distribution, while a high *K* value means a strong binding. In such cases, the life time is longer and the free drug concentration in plasma is lower [31,32].

**Table 2:** Binding constant *K* and number of binding sites *n* for interactions risperidone–HSA and sulpiride–HSA.

Drug	*K* (M^−1^)	*n*
Sulpiride	3.890 (±0.003) × 10^3^	≈1
Risperidone	7.460 (±0.010) × 10^4^	≈1

The above-mentioned strong binding of risperidone could be a consequence of its hydrophobicity, with its metabolizing path by the liver, where it is converted into a hydrophilic metabolite and then eliminated by the kidneys. Otherwise, since 1978, Denisoff and Molle [33] have shown that sultopride, a benzamide derivative like sulpiride, presents reduced tendency to bind to albumins. Sulpiride is a hydrophilic compound, with lower constant values, which presents reduced capacity to entry in the central nervous system and appears to not undergo hepatic action, once no sulpiride metabolite has been identified in plasma, reinforced by the fact that 100% of sulpiride parenteral administration, and 15% to 25% of oral administration are excreted unchanged by the kidneys [13,34,35].

However, despite a different hydrophobicity degree, primary sites for both antipsychotics, sulpiride and risperidone, seem to be located in the same albumin region, within or next to the sub domain IB, where tryptophan residue 134 of BSA is. This subdomain is more exposed to the hydrophilic environment than subdomain IIA, where the tryptophan residue 212 of BSA is. The hydrophobic cavity containing tryptophan residue 134 is less hydrophobic than the subdomain IIA cavity that contains residue 212, which is buried in an internal region of the protein. Sub domain IIA, namely Sudlow I, which normally drives strong hydrophobic interactions involving hydrogen bridges and histidine (residue 242) [36,37].

Thus, results suggest the existence of only one primary binging site for this sulpiride in HSA probably located within or next to the sub domain IB, where the primary site for risperidone also seems to be located. However, the comparison between K-values for two antipsychotics provides evidence that the interaction of albumin to risperidone is very higher than the interaction to sulpiride.

## 3. Material and Methods

### 3.1. Materials

This study was performed *in vitro*, using fluorescence measurements from a Hitachi-F3010 spectrofluorometer (Tokyo, Japan). Albumins (codes A-0281 and A-8763) and sulpiride (S2191000) were purchased from Sigma-Aldrich Co., St. Louis, MO, USA.

### 3.2. Binding of Albumin to Sulpiride—Fluorescence Quenching of Albumin by Spectrofluorimetry

Following the Fragoso *et al.* [16] experimental design, for each quencher addition (from 35.0 to 1191.7 ng/mL increments), we used the wavelength of 290 nm to selectively excite the fluorescence of intrinsic tryptophan residues in albumin. The emission and excitation bandwidths were to 3 nm, and the emission spectra registered from 300 to 400 nm. Final concentrations of sulpiride were within the therapeutic window for psychiatric patients, *i.e.*, from 70.1 to 1121.2 ng/mL, according to Tokunaga *et al.* [38].

For each quenching measurement, sulpiride was added from concentrated stock solution in 2 mL solutions of albumin 2 × 10^−6^ M (in 10 mM phosphate at pH 7.4), at two different temperatures: 25 and 37 °C.

Stern-Volmer graphs were plotted according to the equation$\;F_{0}=F(1+K_{SV}Q)$, where *F_0_* and *F* are the relative fluorescence intensities of albumins in the absence and in the presence of sulpiride respectively (*F_0_* is taken to be 100% always). *K_SV_* is the Stern-Volmer quenching constant, which is related to the bimolecular collisional process, and *Q* is the quencher concentration [17].

The presence of primary and secondary inner filter effects was verified by means of absorbance measurements (Shimadzu UV-160A spectrophotometer, Columbia, Portland, OR, USA) of albumin solutions at emission and excitation wavelengths. The secondary inner filter effect was negligible, and the primary inner filter effect was corrected by Parker’s equation [39]. Comparing Stern-Volmer plots at two temperatures, 25 and 37 °C, we verified the dynamic or static nature of the quenching process but only for HSA.

Regression coefficients in Stern-Volmer analysis allow us to fit a linear adjust when *r*^2^ > 0.991 and *p* < 0.0001. In the case where *r*^2^ < 0.9980, the polynomial adjust was used [16].

The constant of binding (*K*) and the number of binding sites (*n*) of sulpiride to HSA were obtained through the equation [25]

(1) $$log\left(\frac{F_{0}-F}{F}\right)=log\left(K\right)+n\;log([Q])$$

### 3.3. Mathematical Modeling of Albumin Quenching by a Fluorescent Quencher

Due to the fluorescence of the sulpiride molecule, which difficult the direct determination of the quenching of the albumin, a mathematical model has been developed aiming to distinguish the albumin fluorescence from the sulpiride fluorescence, in the solution where they interact.

For a certain concentration of ligand *i* added in the solution, we can consider that the fluorescence measured for albumin, F*_i_*, at a given temperature can be written as

(2) $$F_{i}=F_{A_{0}}+F_{L_{0}}-F_{S_{T}}$$

where $F_{A_{0}}$ is the fluorescence intensity of albumin before the addition of the quencher, $F_{L_{0}}$ is the fluorescence intensity of the pure quencher solution, and $F_{S_{T}}$ is the total fluorescence quenched due to the interaction of those molecules. $F_{S_{T}}$ can be represented by the sum of the quenched fluorescence portion of albumin $F_{A_{S}}$ and the quenched fluorescence portion of the quencher $F_{L_{S}}$, *i.e.*, $F_{S_{T}}=F_{A_{S}}+F_{L_{S}}$.

Supposing that each chemical species contributes with half of the quenched fluorescence when interacting, we can write that $F_{S_{T}}=2F_{A_{S}}$. Substituting Equation (4) into Equation (2), the fluorescence intensity involved in the interaction is given by $F_{A_{S}}=\frac{1}{2}(F_{A_{0}}+F_{L_{0}}-F_{i})$. Subtracting $F_{A_{0}}\;$from both sides of this equation and dividing them by $F_{A_{0}}$, we have

(3) $$\frac{F_{A_{i}}}{F_{A_{0}}}=\frac{1}{2}-\frac{F_{L_{0}}}{2F_{A_{0}}}+{\frac{F_{i}}{2F_{A_{0}}}}_{\;}$$

which refers to the recorded fluorescence portion emitted by albumin. This equation is valid for all fluorescence measures, from the beginning of the titration of albumin solution by a fluorescent quencher, such as sulpiride.

## 4. Conclusions

Results support the conclusion that sulpiride quenches HSA and BSA fluorescence by a process of static quenching, interacting with the protein by forming complexes. Association constants calculated for sulpiride/HSA <0.6 µM, by the Stern-Volmer equation, were 2.20 (±0.08) × 10^4^ and 5.46 (±0.20) × 10^4^ M^−1^, at 37 and 25 °C, respectively. For sulpiride–BSA interaction, association constants were 0.44 (±0.01) × 10^4^ and 2.17 (±0.04) × 10^4^ M^−1^ at 37 and 25 °C, respectively.

The apparent binding constant estimated for sulpiride–HSA at 37 °C was 3.89 (±0.03) × 10^3^ M^−1^, which was lower than that found by Fragoso *et al.* [16] to risperidone-HSA at 37 °C.

The comparison between *K*_sv_ values allows us to conclude that the quenching potential of RPD on HSA at 37 °C is about six times higher than that for sulpiride. Despite different hydrophobicity degrees, primary sites for both antipsychotics appear to be located in the same albumin region, within or next to the sub domain IB, where tryptophan residue 134 of BSA is located, the less hydrophobic between both considered regions.

The biological meaning of this study lies in its contribution to the knowledge about the pharmacokinetics of sulpiride, and its comparison with risperidone. It is of great relevance in some clinical pharmacology and psychiatric questions to rationalize and personalize therapeutics. In such a sense, the simultaneous administration of drugs and therapeutics requires critical care due to their possible competition for the same site.

In addition, the present study contributes to the molecular biochemistry of sulpiride, establishing the number of binding sites, found to be only one, and its localization in the chain.
